# α-Klotho Expression in Human Tissues

**DOI:** 10.1210/jc.2015-1800

**Published:** 2015-08-17

**Authors:** Kenneth Lim, Arnoud Groen, Guerman Molostvov, Tzongshi Lu, Kathryn S. Lilley, David Snead, Sean James, Ian B. Wilkinson, Stephen Ting, Li-Li Hsiao, Thomas F. Hiemstra, Daniel Zehnder

**Affiliations:** Renal Division (K.L., T.L., L.-L.H.), Brigham and Women's Hospital, Harvard Medical School, Boston, Massachusetts 02115; Cambridge Centre for Proteomics (A.G., K.S.L., T.F.H.), and School of Clinical Medicine (I.B.W., T.F.H.), University of Cambridge, Cambridge CB2 0QQ, United Kingdom; Department of Pathology (D.S., S.J., S.T.), University Hospitals Coventry and Warwickshire, NHS Trust, Coventry CV2 2DX, United Kingdom; and Division of Translational Research (G.M., D.Z.), Warwick Medical School, University of Warwick, Coventry CV2 2DX, United Kingdom

## Abstract

**Context:**

α-Klotho has emerged as a powerful regulator of the aging process. To date, the expression profile of α-Klotho in human tissues is unknown, and its existence in some human tissue types is subject to much controversy.

**Objective:**

This is the first study to characterize systemwide tissue expression of transmembrane α-Klotho in humans. We have employed next-generation targeted proteomic analysis using parallel reaction monitoring in parallel with conventional antibody-based methods to determine the expression and spatial distribution of human α-Klotho expression in health.

**Results:**

The distribution of α-Klotho in human tissues from various organ systems, including arterial, epithelial, endocrine, reproductive, and neuronal tissues, was first identified by immunohistochemistry. Kidney tissues showed strong α-Klotho expression, whereas liver did not reveal a detectable signal. These results were next confirmed by Western blotting of both whole tissues and primary cells. To validate our antibody-based results, α-Klotho-expressing tissues were subjected to parallel reaction monitoring mass spectrometry (data deposited at ProteomeXchange, PXD002775) identifying peptides specific for the full-length, transmembrane α-Klotho isoform.

**Conclusions:**

The data presented confirm α-Klotho expression in the kidney tubule and in the artery and provide evidence of α-Klotho expression across organ systems and cell types that has not previously been described in humans.

The identification of the novel anti-aging protein α-Klotho in 1997 (1) first challenged the long-held paradigm of aging as a passive, inevitable process of deteriorating organ function and declining health. Because α-Klotho knockout mice exhibited a shortened life span and transgenic mice that overexpress α-Klotho live 30% longer (2), aging has instead emerged as a regulated and potentially modifiable process. α-Klotho deficiency results in a variety of features characteristic of mammalian aging including organ atrophy, infertility, vascular calcification, atherosclerosis, osteomalacia, osteoporosis, peripheral insulin sensitivity, metabolic derangements, and cerebral changes (1), all of which occur in “normal” aging.

In humans, emerging data indicate that aging is also modifiable and subject to regulation by complex genomic, proteomic, and environmental interactions (3). Premature or accelerated aging occurs in a number of human genetic disorders such as Werner syndrome and Hutchinson-Gilford progeria syndrome, conditions that recapitulate many or all of the features of normal aging. Furthermore, features of aging and a reduced life span accompany a number of chronic disease states in humans, including chronic kidney disease, cancer, diabetes, HIV, and inflammatory arthropathies (4). Only one case of a human α-Klotho mutation has been described to date, but polymorphisms in the α-Klotho gene (KL-VS variant) are associated with normal human aging (5). Given the implications of these discoveries for human health, there has been much interest in molecules such as α-Klotho as potential longevity-modulating therapeutic targets.

α-Klotho has two known human isoforms. The full-length protein is a 130-kDa, 1012 amino acid (AA), single-pass transmembrane protein that contains a signal sequence, two homologous domains (denoted KL1 and KL2), a transmembrane domain, and a short cytoplasmic tail. The second isoform (62 kDa, 549 AA) arises from alternative splicing and is a secreted soluble protein (hereafter referred to as ^S^α-Klotho) that contains only the SS domain and KL1, with the terminal 15 residues replaced by SQLTKPISSLTKPYH (Figure 1A) (5). Although the secreted isoform predominates and circulates in plasma, its function is largely unknown. In contrast, full-length α-Klotho is involved in aging and in phosphate homeostasis. α-Klotho functions as a coreceptor with the fibroblast growth factor (FGF) receptor for the phosphatonin FGF23. Additional pleiotropic functions have been ascribed to tissue α-Klotho, including protection against oxidative stress (6), inhibition of apoptosis (7) and fibrogenesis (8), promotion of angiogenesis and vascularization (9), vasculoprotective properties (10, 11), and regulation of stem cell proliferation through modulation of Wnt signaling (12), all of which may protect against aging.

**Figure 1.**
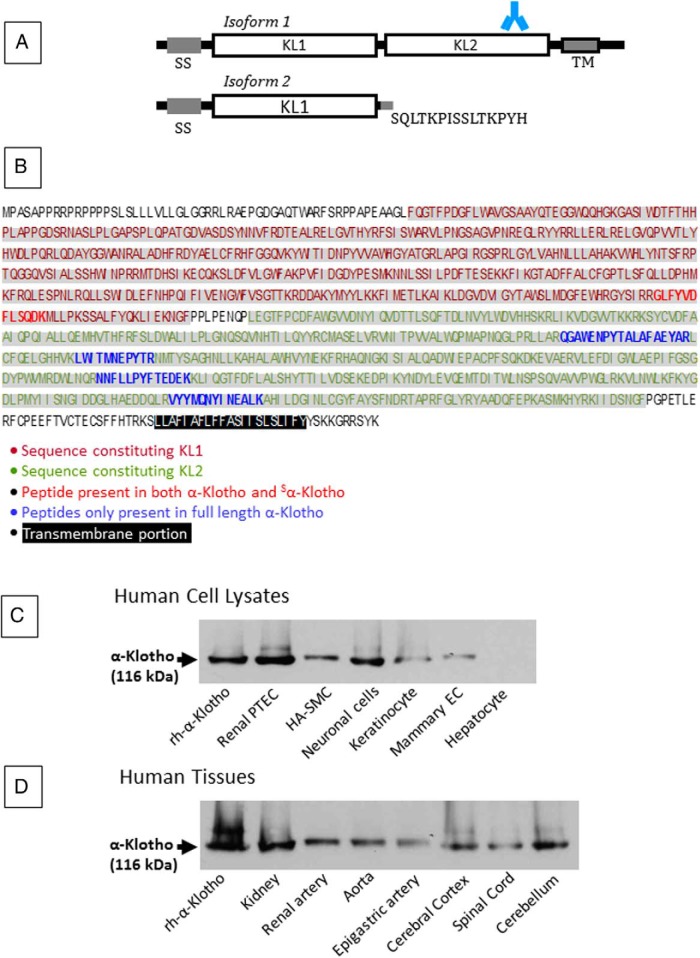
α-Klotho isoforms, sequence, and Western blot. A, Structure of the two isoforms of α-Klotho. Isoform 1 represents the full-length protein and contains a signal sequence domain (SS), two homologous domains (KL1, KL2), a short transmembrane domain (TM), and a short cytoplasmic tail. Shown is the site of the epitope for the antibody used in our experiments: AA 800 to 900, KL2. This epitope is absent from Isoform 2, a soluble, secreted protein that arises from alternative RNA splicing and contains only AA 1–549, and where the terminal 15 residues are replaced by the sequence shown. B, The full-length α-Klotho protein sequence of 1012 AA is shown, with KL1 and KL2 shown in red and green respectively, and TM highlighted (black). The peptides giving rise to the PRM signature are also shown (bold typeface; common to isoforms 1 and 2, red; exclusive to full-length α-Klotho, isoform 1, blue). C and D, Western blot analysis of cell lysates (C) and tissues (D) supports the presence of the full-length α-Klotho. Full-length rh α-Klotho protein (rh-α-Klotho). EC, epithelial cells.

α-Klotho protein expression has been found in mice, rats, and humans predominantly in the renal distal convoluted tubular cells (1) and to a lesser extent in proximal convoluted tubular epithelial cells (PTECs) (13) and the parathyroid gland (14). We recently reported α-Klotho expression in human vasculature (11). Although α-Klotho expression has been described in other rodent tissue types, including the pituitary gland, pancreas, ovary, testis, placenta, choroid plexus of the brain (1). and most recently in rat aorta (15), no study to date has systematically investigated α-Klotho expression in humans. Expression in some tissues, particularly the cardiovascular system, has been controversial (16), partly due to limitations and variability of antibody-based techniques employed to date that may not distinguish between α-Klotho and ^S^α-Klotho. Given its implications for human health, data on the expression of α-Klotho in human tissues are urgently needed.

To overcome the above limitations, we employed state-of-the-art targeted proteomic analysis using parallel reaction monitoring (PRM) in parallel with conventional antibody-based methods to determine the distribution and precise nature of human α-Klotho expression in health.

## Materials and Methods

### Tissue sample and preparation

Cell lysates were obtained from commercially available primary cell cultures (ScienCell Research Laboratories), including: Human Epidermal Keratinocyte Lysate-adult (product code HEKL-a, catalog no. 2116), Human Prostate Epithelial Cell Lysate (product code HPrEpiCL, catalog no. 4416), Human Mammary Epithelial Cell Lysate (product code HMEpiCL, catalog no. 7616), Human Renal Proximal Tubular Epithelial Cell Lysate (product code HRPTEpiCL, catalog no. 4106), and Human Neuron Lysate (product code HNL, catalog no. 1526). Human hepatocytes (HepG2; Sigma) were kindly gifted by Dr. Graeme Alexander, University of Cambridge. Human aortic smooth muscle cells (HA-SMCs) were provided by Caltag Medsystems (catalog no. SC-6110; ScienCell).

Human tissues for immunohistochemistry (IHC) and tissue lysate preparation were obtained with local ethical approval and informed written consent from four sources: 1) surgical specimens from the human tissue bank, Department of Pathology, University Hospital Coventry, and Warwickshire NHS Trust, UK (ethics approval, 13\WM\0072); 2) artery and kidney tissue from Warwick Medical School, UK (ethics approval, 05/Q2802/26 and 10/H12111/36); 3) aorta from the Department of Medicine, University of Cambridge (ethics approval, 05/MRE04/7); and 4) neuronal tissue, kindly donated by the New York Brain Bank at Columbia University, New York. Human parathyroid whole cell lysate was obtained from Abcam (catalog no. ab29792).

### IHC for α-Klotho protein

The rabbit polyclonal α-Klotho antibody (catalog no. Ab69208 and Ab181373; Abcam) and rabbit polyclonal isotype antibody (catalog no. Ab27478) were used at a concentration of 1:100 to 1:250 for IHC staining and control experiments on formalin-fixed and paraffin-embedded, glass-mounted sections. Please see Supplemental Data for details of the IHC protocol.

Assessment of α-Klotho distribution along the nephron was done morphologically. Determination was limited to proximal tubular and distal tubular (distal tubule and collecting duct) nephron segments. Consecutive section staining with hematoxylin and eosin was also used.

### Tissue preparation for Western blot and mass spectrometry

Human tissues were homogenized in liquid nitrogen and resuspended in standard RIPA buffer supplemented with protease inhibitors cocktail (Sigma) and 1% Triton X-100. The lysates were clarified by microcentrifugation at 10 000 × *g* for 10 minutes at 4°C, and protein concentration was assayed by Lowry assay. As a control, we used recombinant human (rh) full-length α-Klotho protein (rh-α-Klotho) (5334-KL-025; R&D Systems).

### Western blot for α-Klotho protein

Aliquots of cell lysates containing 20 μg protein were separated by SDS-PAGE and Western blotted with antihuman α-Klotho (catalog no. Ab69208; Abcam) at a concentration of 1:1000. Ten micrograms of rh-α-Klotho were used as control (see Supplemental Data). The expected protein size for the full-length α-Klotho protein was 116 kDa.

### Sample preparation for mass spectrometry

All protein samples (50 μg) were fractionated on precast SDS-PAGE gels (4–15%, 10 wells; catalog no. 456–1083; BioRad) and stained with Coomassie blue stain. All samples were run with either an empty lane in between or on separate gels to avoid sample-to-sample contamination.

To restrict analyses to the full-length protein, only the gel band between 75 and 150 kDa was resected for analysis, thus excluding ^S^α-Klotho (Figure 1A). Gel fractions were macerated with a sterile blade and subjected to in-gel digestion. Gel fractions were destained by three washes with 80 μL of 50% acetonitrile (ACN)/50 mm ammonium hydrogen carbonate and washed with 100% ACN. The alkylation step was omitted, given the absence of cysteine residues from target α-Klotho peptides. Tryptic digestion was carried out overnight at 37°C with 60 μL of trypsin (sequencing grade modified, catalog no. V511A; Promega) in 50 mm ammonium hydrogen carbonate (0.005 μg/μL). This process yielded 25 μL, of which 5 μL (1 μL for rh-α-Klotho) was subjected to liquid chromatography-electrospray ionization tandem mass spectrometry (MS/MS) and PRM analysis in an Orbitrap nano-ESI Q-Exactive mass spectrometer (Thermo Scientific), coupled to a nanoLC (Dionex Ultimate 3000 UHPLC).

### Data-dependent acquisition and PRM

A top 10 data-dependent acquisition MS/MS analysis was performed first to assess sample quality. Samples were trapped on a 100 μm × 2 cm, C18, 5 μm, 100 trapping column (Acclaim PepMap 100) in μL-pickup injection mode at 4 μL/min flow rate for 10 minutes. Samples were then loaded on a Rapid Separation Liquid Chromatography, 75 μm × 50 cm nanoViper C18 3 μm 100 column (Acclaim, PepMap) retrofitted to an EASY-Spray source with a flow rate of 350 nL/min (buffer A, HPLC H_2_O, 0.1% formic acid; buffer B, 100% ACN, 0.1% formic acid; 60-min gradient; 0–5 min: 5% buffer B, 5–45 min: 5 to >56% buffer B, 45.1 to 50 min: 56% to >95% buffer B, 50.1 to 60 min, 5% buffer B). Peptides were transferred to the gaseous phase with positive ion electrospray ionization at 1.8 kV. In data-dependent acquisition, the top 10 precursors were acquired between 400 and 1600 m/z with a 2Th (Thomson) selection window, dynamic exclusion of 30 seconds, normalized collision energy of 25, and resolution of 70 000. For PRM, precursors were targeted in a 2Th selection window around the m/z of interest. Precursors were fragmented in high-energy collisional dissociation mode with normalized collision energy dependent on the target peptide. The first mass analysis was performed at a 70 000 resolution, an automatic gain control target of 3e (6), and a maximum C-trap fill time of 200 milliseconds; MS/MS was performed at 35 000 resolution, an AGC target of 5e (4), and a maximum C-trap fill time of 100 milliseconds. Spectra were analyzed using Skyline and/or MASCOT, with manual validation. A peptide was considered present when at least one fragment ion was detected by Skyline, in addition to the identification of a clear isotope pattern of the fragment ion in the raw data, at the expected retention time for the parent peptide.

### Data processing and statistical analysis

Skyline analysis utilized .raw Thermo files. For MASCOT searches, .mgf files were generated from .raw Thermo files using MSconvert, uploaded in MASCOT, and searched against a human database (Uniprot 2013) using the following settings: carbamidomethyl as fixed modification, methionine oxidation as variable modification; 25 ppm peptide tolerance, 0.8 Da MS/MS tolerance; maximum of two missed cleavages, peptide selection charge of +2, +3, or +4, and selection of a decoy database.

## Results

### Spatial distribution of α-Klotho protein in human tissues

**Figure 2.**
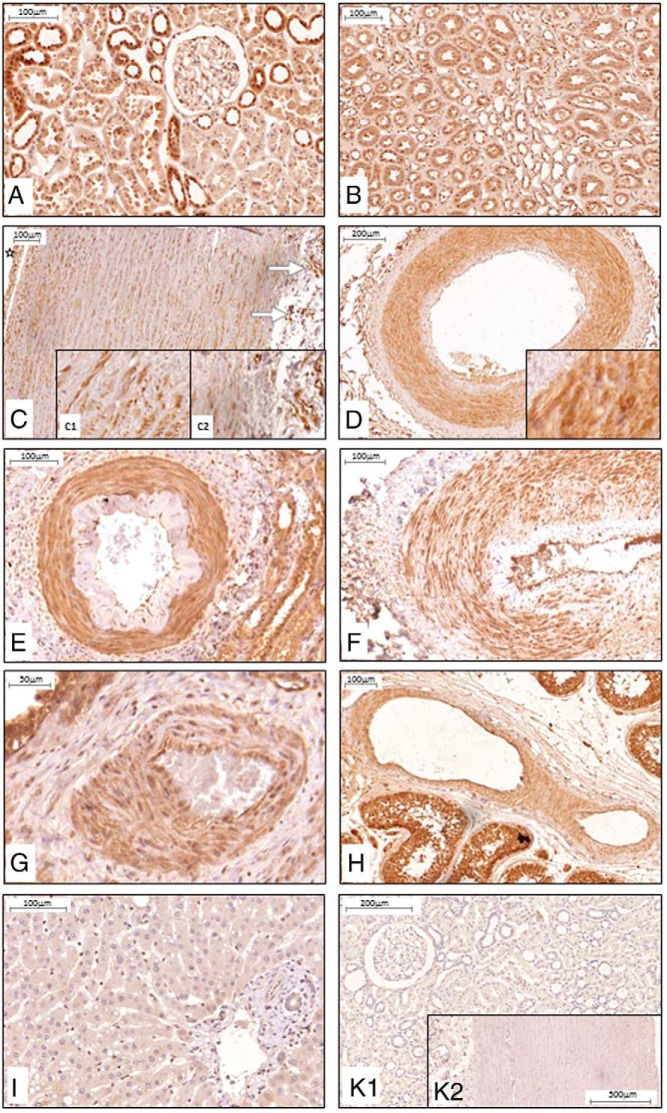
α-Klotho protein expression and distribution in human kidney, artery, and liver. IHC, positive staining (brown) was found in tubular cells of the kidney cortex (A), with variable expression in proximal and stronger expression in distal tubules; medulla (B); the smooth muscle cell layers of artery such as aorta (C) with staining of intima and media (C1) and stronger staining of the vasa vasorum (arrows, C2); and lumen indicated by star. D, Renal artery. E, Intrarenal artery, here with intimal changes probably due to hypertensive vascular changes, F–G, Artery in thyroid (F), prostate (G). and testis (H). I, Hepatocytes did not show any positive staining for α-Klotho protein. K, Negative kidney tissue control (K1) and aorta (K2) with isotype antibody control. n ≥ 5 for each tissue.

Consistent with previous reports (13), we confirmed expression of α-Klotho in both the proximal and distal tubular epithelial cells of the kidney by IHC (Figure 2, A and B). In subsequent experiments, kidney tissue served as a positive control.

### α-Klotho is expressed throughout the arterial tree

Expression of α-Klotho was identified in smooth muscle cells throughout the arterial tree (Figure 2, C–H). In proximal vessels, α-Klotho was present in elastic (aorta; Figure 2C) and muscular (renal; Figure 2D) artery. Aorta α-Klotho was expressed to a similar level in intima and media layer (Figure 2C, inset C1) but was much stronger in the vasa vasorum of the adventitia (Figure 2C, inset C2). More distally, α-Klotho was also identified in smaller arteries within vascular beds in kidney, thyroid, prostate, and testis (Figure 2, E–H). We next sought α-Klotho in the liver, but α-Klotho could not be identified in hepatocytes (Figure 2I).

### α-Klotho is present in epithelia

**Figure 3.**
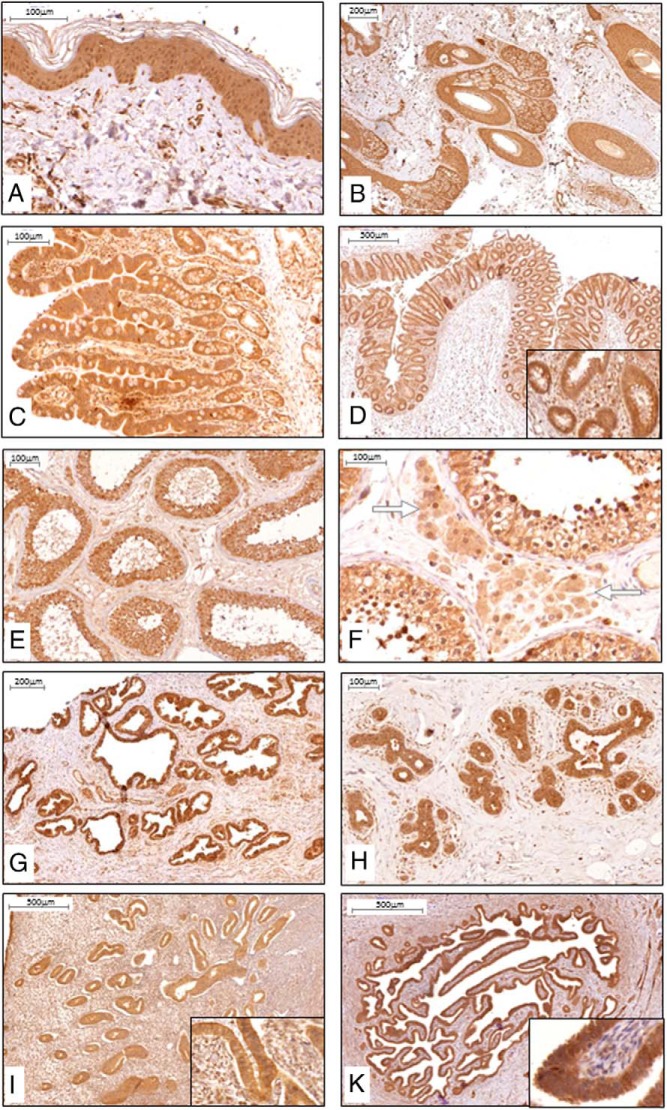
α-Klotho protein expression and distribution in human epithelial and reproductive tissues. IHC, positive staining (brown) was found in all the cellular layers of the epidermis (A) and appendage tissue such as hair follicle and sebaceous gland (B). Intestinal expression was primarily found in epithelial cells as illustrated in jejunum (C) and colon (D). In reproductive tissues, positive staining was found in epithelial Sertoli cells (E), testosterone producing Leydig cells (illustrated with white arrows) of the testis (F), and epithelial cells of the prostate gland G). H–K, In mammary tissue (H), endometrium of uterus (I), and endometrium of salpinx (K), the epithelial cell layer was staining strongly for α-Klotho protein; insets are larger magnifications of the epithelial layer. n ≥ 5 for each tissue.

We next examined α-Klotho protein expression in epithelial tissues. α-Klotho was highly expressed throughout the epidermis (Figure 3A) and skin appendages such as hair follicles and sebaceous glands (Figure 3B). In addition, epithelial cells of both the small bowel (jejunum; Figure 3C) and colon (Figure 3D) also demonstrated strong expression of α-Klotho.

In the male reproductive system, α-Klotho was expressed in Sertoli cells (Figure 3, E and F) of the testis, as well as epithelial cells of the prostate gland (Figure 3G). In the female reproductive system, α-Klotho expression was present in mammary epithelium (Figure 3H), endometrium of the uterus (Figure 3I), and salpynx (Figure 3K).

### α-Klotho is present in endocrine tissues

**Figure 4.**
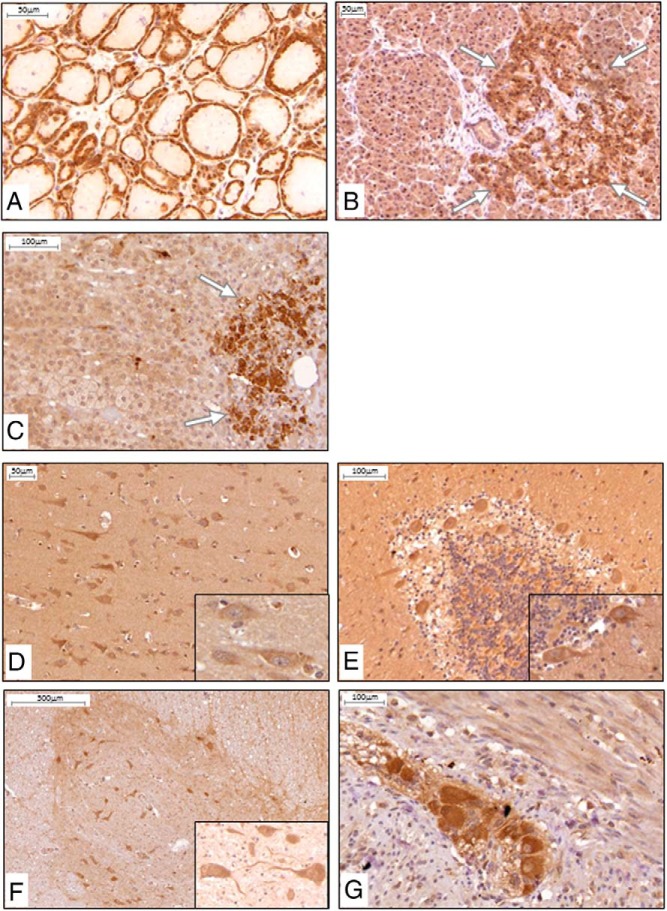
α-Klotho protein expression and distribution in human endocrine and neuronal tissues: IHC, positive staining (brown) was found in principal cells of the thyroid epithelium (A), islet cells of the pancreas (B), and catecholamine-producing medullary cells of the adrenal gland (C). When brain and spinal cord were stained, primarily neuronal cells were found to express α-Klotho protein, such as in the cerebral cortex (D), Purkinje cells between the molecular and granular layer in the cerebellum (E), motoneurons in the gray matter of the ventral horns of the spinal cord (F), and neuron cell bodies in the ganglia of the myenteric plexus of the intestine (G). Insets are larger magnifications of neuronal cell bodies. n ≥ 5 for each tissue.

Examination of endocrine tissue revealed α-Klotho expression in follicular epithelial cells of the thyroid (Figure 4A), in the insulin-producing islet cells of the pancreas (Figure 4B), in medullary cells of the adrenal gland (Figure 4C), and in testosterone producing Leydig cells (Figure 3F) of the testis.

### α-Klotho is present in neuronal cells

We next examined α-Klotho expression in neural tissues. Expression was found in neuronal cells of the cerebral cortex (Figure 4D). In the cerebellum, α-Klotho was expressed primarily in Purkinje cells between the molecular and granular layer (Figure 4E). In the spinal cord, α-Klotho was present in the motor neurons in the gray matter of the ventral horn (Figure 4F). In the myenteric plexus, α-Klotho protein was found in ganglial neuronal cell bodies (Figure 4E).

### Analysis of α-Klotho expression by Western blotting

To confirm the presence and molecular weight of α-Klotho for those tissues where it was identified by IHC experiments, we next performed Western blotting on both human tissue lysates and (where available) human primary cells. For these experiments, we used full-length rh-α-Klotho protein as positive control. Western blotting demonstrated the presence of full-length (116 kDa) α-Klotho in lysates from human renal PTEC, HA-SMCs, neuronal cells, keratinocytes, and mammary epithelial cells (Figure 1C). Consistent with IHC (Figure 2I), α-Klotho could not be identified in hepatocytes. In human tissue lysates, full-length α-Klotho was identified at 116 kDa in kidney, renal artery, aorta, epigastric artery, cerebral cortex, spinal cord, and cerebellum (Figure 1D).

### Mass spectrometry analysis of human samples

The data described above provide strong support for the presence of full-length α-Klotho in the tissues shown. However, because these data are antibody-dependent, we next sought to corroborate these findings using PRM, a targeted proteomics approach. PRM is able to robustly and reproducibly identify a peptide signature for a protein of interest with precision and specificity and, importantly, can yield isoform-specific data. This methodology is therefore able to distinguish ^S^α-Klotho from α-Klotho. To further mitigate against the potential for contamination with ^S^α-Klotho, samples were first fractionated by one-dimensional gel electrophoresis. Only gel fragments between 75 and 150 kDa were excised for analysis, thus excluding the ^S^α-Klotho isoform (62 kDa). Full-length transmembrane rh-α-Klotho protein served as control and allowed generation of a reference PRM signature.

**Figure 5.**
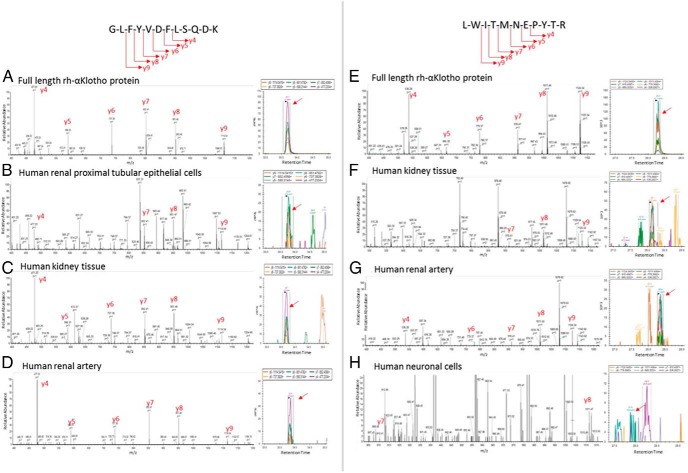
Mass spectrometry characterization of the transmembrane α-Klotho protein in human tissues and cells: extracellular α-Klotho peptide GLFYVDFLSQKD (exon 3). A–D, Representative mass spectrometry spectra (left) and Skyline data (right) confirmed the presence of full-length α-Klotho rh full-length α-Klotho protein (rh-α-Klotho) (A), renal proximal tubular epithelial cells (B), kidney tissue (C), and renal artery (D). E–G, The full-length specific (isoform 1) αKlotho peptide LWITMNEPYTR (exon 4). Representative mass spectrometry spectra (left) and Skyline data (right) confirmed the presence of full-length α-Klotho. E), rh full-length α-Klotho protein (rh-α-Klotho); F, kidney tissue; G, renal artery; and H, neuronal cells.

First, spectra for 16 candidate peptides unique to α-Klotho were generated by subjecting purified rh-α-Klotho to liquid chromatography-electrospray ionization MS/MS (Supplemental Table 1). We next sought the presence of these peptide spectra in positive control samples (human kidney and PTEC lysates) by PRM, aiming to identify the most reliable spectra and seeking in particular peptide spectra present in α-Klotho but absent in ^S^α-Klotho. The most robust signatures were yielded by GLFYVDFLSQDK (α-Klotho and ^S^α-Klotho) and QGAWENPYTALAFAEYAR, NNFLLPYFTEDEK, VYYMQNYINEALK, and LWITMNEPYTR (all absent from ^S^α-Klotho). Given that GLFYVDFLSQDK is also present in ^S^α-Klotho, it does not distinguish between isoforms; in contrast, QGAWENPYTALAFAEYAR, NNFLLPYFTEDEK, VYYMQNYINEALK, and LWITMNEPYTR are not present in ^S^α-Klotho and can only be identified if full-length α-Klotho is present. We therefore restricted subsequent PRM analyses to GLFYVDFLSQDK and the four full-length-specific peptides described above. Figure 5, A–H, shows representative spectra for GLFYVDFLSQDK and LWITMNEPYTR from rh-α-Klotho, human kidney lysate, and human PTEC lysate.

We next sought the presence of these peptide signatures in a variety of human tissue and cell lysates. α-Klotho extracellular domain peptide GLFYVDFLSQDK was present in whole kidney lysate, kidney cortex, kidney medulla, proximal tubular epithelial cells, parathyroid, pancreas, keratinocytes, mammary epithelial cells, prostate epithelial cells, neuronal cells, cerebral cortex, cerebellum, and artery (aorta, renal, and epigastric artery) (Table 1). The spectrum for GLFYVDFLSQDK in human renal artery is shown (Figure 5D) as a representative example.

**Table 1.** Confirmation of Transmembrane α-Klotho Protein Expression in Human Tissues and Cells

	Peptide of Extracellular Portion	Peptide of Transmembrane Portion
Human cells		
Recombinant human α-Klotho protein	+	+
Kidney proximal tubular epithelial cells	+	+
Keratinocyte	+	+
Mammary epithelial cells	+	−
Prostate epithelial cells	+	−
Neuronal cells	+	+
Human tissue		
Recombinant human α-Klotho protein	+	+
Kidney	+	+
Kidney cortex	+	+
Kidney medulla	+	+
Parathyroid gland	+	+
Pancreas	+	+
Cerebral cortex	+	+
Cerebellum	+	+
Aorta	+	+
Muscular artery	+	+

PRM-based targeted mass spectrometry was used to characterize the presence of peptide fragments from the extracellular and transmembrane peptide portions.

Peptides present only in the full-length α-Klotho and absent from ^S^α-Klotho were identified in whole kidney lysate, kidney cortex, kidney medulla, proximal tubular epithelial cells, parathyroid, pancreas, keratinocytes, neuronal cells, cerebral cortex, cerebellum, and artery (aorta, renal, and epigastric artery), indicating the presence of full-length α-Klotho in these tissues (Table 1). Representative spectra for LWITMNEPYTR in human renal artery and human neuronal cells are shown in Figure 5, G and H. Detailed peptide evidence for full-length α-Klotho in each sample is shown in Supplemental Table 2. All proteomic data have been deposited with the ProteomeXchange consortium open access repository and can be accessed at http://proteomecentral.proteomexchange.org (identifier PXD002775).

## Discussion

We report the identification of full-length α-Klotho protein in a wide variety of human tissues including the arterial tree, epithelia, endocrine, and neuronal tissues. Previously, α-Klotho protein expression in humans had only been described in the kidney (13) and parathyroid glands (14), along with our own report of α-Klotho in human muscular artery (11). Our findings are consistent with data from rodent studies demonstrating α-Klotho expression in pituitary gland, pancreas, ovary, testis, placenta, and choroid plexus of the brain (1) and suggest tissue-specific roles for α-Klotho at sites not involved in phosphate transport signaling.

The wide tissue distribution of α-Klotho is consistent with its known role in aging. Rodent models of α-Klotho deficiency demonstrate reduced life span with a wide range of tissue phenotypes including gonadal failure, arteriosclerosis, emphysema, impaired cognition, hearing loss, vascular calcification, cardiac hypertrophy, osteopenia, and atrophy of skin, adipose tissue, thymus, and skeletal muscle (1). In humans, α-Klotho deficiency or functional variants of α-Klotho are associated with the development of vascular calcification (11, 17), atherosclerosis (18), diabetes (19), hypertension (20), chronic kidney disease (21), osteoporosis (22), anemia (23), and various cancers such as hepatocellular carcinoma (24), breast cancer (25), gastric cancer (26), and renal cell carcinoma (27).

Our data show full-length α-Klotho in elastic and muscular artery, consistent with our previous report (11). Other investigators have failed to demonstrate the presence of α-Klotho in vascular tissue (16). This apparent conflict is most likely due to differences in sample preparation, experimental conditions, and antibodies employed, and it highlights the difficulties inherent in antibody-based methods. Furthermore, given the hydrophobic nature of full-length α-Klotho, its isolation from experimental samples is not straightforward. Here, data derived from PRM experiments demonstrate the presence of full-length α-Klotho in human artery. Given that α-Klotho deficiency results in a striking arterial phenotype (1) and is associated with an increased risk of coronary artery disease (28) and stroke (29), our data confirming the presence of α-Klotho in the human artery tree are of high clinical importance.

Expression of α-Klotho in human skin has not previously been reported. α-Klotho deficiency results in skin atrophy, a hallmark of the aging process. The mechanisms by which α-Klotho maintains healthy skin are not known. We also identified α-Klotho in other epithelia including the intestine. Although it is possible that α-Klotho may similarly maintain intestinal epithelial health, it may equally be involved in phosphate and calcium transport and homeostasis. Indeed, α-Klotho may regulate expression of intestinal TRPV5 and TRPV6 as observed in kidney (30). Evidence for this has been shown in animal models examining the role of Klotho-FGF23 in regulating phosphate concentration via NaPi-II transporters in intestinal epithelial cells (31) and renal distal tubules (32).

We found strong expression of α-Klotho in thyroid follicular cells, suggesting that it may be involved in the regulation of thyroid hormone production. Given that clinical and subclinical hypothyroidism increases in prevalence with increasing age, α-Klotho may be involved in regulating synthesis of thyroid hormone (33). This may similarly be true for testosterone synthesis from Leydig cells, which also declines with age. Indeed, Hsu et al (34) have proposed the existence of a testosterone α-Klotho feedback loop. In our study, α-Klotho expression was also found in insulin-producing islet cells of the pancreas. This finding assumes considerable significance given emerging evidence that demonstrates a role for α-Klotho in regulating the insulin/IGF-1 pathway, a highly conserved mechanism that itself impacts on life span (2, 35). Finally, α-Klotho was also found in the adrenal medulla, the site of synthesis of catecholamines, hormones that regulate the immediate stress response, with a significant role in the cardiovascular system.

We identified α-Klotho at various sites throughout the central nervous system (CNS). Whereas previous reports have demonstrated α-Klotho in the rodent brain choroid plexus, Purkinje cells of the cerebellum (36), and studies in humans confirm the presence of α-Klotho in cerebrospinal fluid in health, its CNS tissue distribution has not previously been described in humans (37, 38). The functional significance of α-Klotho in the CNS remains largely unknown. However, reduced cerebrospinal fluid α-klotho was found to be associated with age and the development of Alzheimer's disease in one small cohort of 70 patients (38). Intriguingly, one recent report by Dubal et al (39) demonstrated that the KL-VS functional variant of the α-Klotho gene was associated with enhanced cognition in three independent cohorts of healthy older humans. These observations suggest that α-Klotho may modulate human learning and memory and may be protective against degenerative disease within the CNS.

Although we identified α-Klotho in a wide variety of organs and tissues, it could not be identified in hepatocytes. This is consistent with a recent report by Chen et al (40), showing that although α-Klotho is increasingly expressed with progressive dedifferentiation in hepatoma cells, normal liver was negative for α-Klotho staining on IHC. In addition, hepatocytes (including HepG2 cells used in our experiments) did not show α-Klotho in Western blot experiments. Although α-Klotho appears to be absent, an analogous system is operational in hepatocytes; the α-Klotho homolog, β-klotho, is expressed in hepatocytes and adipose tissue, where it appears to have a role in glucose metabolism. Similar to α-Klotho which forms a coreceptor with the FGF receptor for FGF23 in phosphate signaling, β-klotho complexes with FGF receptors in hepatocytes to form a coreceptor for FGF19 and FGF21 (41).

Our study has several important strengths. First, our report is the first to systematically evaluate α-Klotho expression in a wide range of human tissue samples and cell types. Second, we have confirmed our previous observation of α-Klotho in the vascular tree. Third, in parallel to IHC and Western blotting, we have employed state-of-the-art PRM to provide antibody-independent data confirming the presence of α-Klotho within samples. All mass spectrometry data are publically accessible. Furthermore, we were able to identify peptide signatures specific for isoform 1 of α-Klotho (Figure 1), allowing us to demonstrate with a high degree of certainty the presence of full-length transmembrane α-Klotho. This finding cannot be explained by tissue contamination with soluble exogenous α-Klotho.

Our findings should be interpreted against the limitations of our study. First, it has proven very challenging to liberate the hydrophobic full-length α-Klotho protein sufficiently to allow detection. Although evidence of presence is robust, the absence of peptides derived exclusively from full-length α-Klotho (isoform 1) should be interpreted with caution, for example in the case of prostate and mammary epithelial cell lines (Table 1). Secondly, any study of human tissues is limited by the availability of experimental material, and our panel of samples and cell lines was not exhaustive.

In summary, this is the first study to systematically characterize tissue expression of full-length α-Klotho in humans. Given the influence of α-Klotho on longevity and the impact of its deficiency on health and aging across multiple organ systems, our data provide an important first step toward elucidating the role of α-Klotho in health and disease.
